# Correction: ARFRP1 functions upstream of ARL1 and ARL5 to coordinate recruitment of tethering factors to the trans-Golgi network

**DOI:** 10.1083/jcb.20190509710072019c

**Published:** 2019-10-10

**Authors:** Morié Ishida, Juan S. Bonifacino

Vol. 218, No. 11, November 4, 2019. 10.1083/jcb.201905097.

The authors noticed an error in the scheme shown in Fig. 8 of their manuscript. Two arrows pointing from ARFRP1 to ARL1 through GEF were in the wrong direction. The mistake in this model figure has now been corrected; the revised Fig. 8 is shown below. All versions of the article have been corrected. This error appears only in PDF versions downloaded on or before October 10, 2019.

Rockefeller University Press

**Figure 8.**
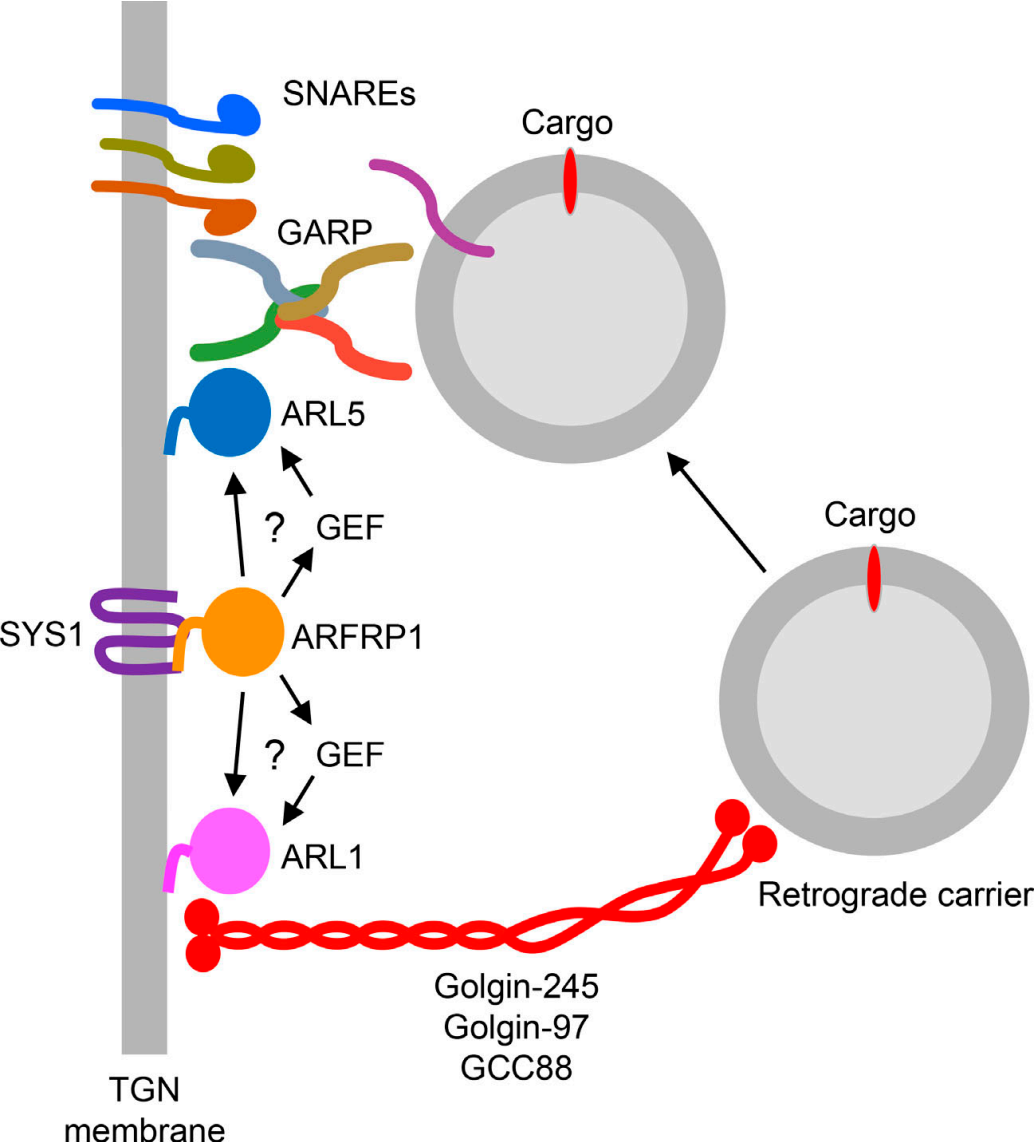
**Model for the function of ARFRP1 in the coordinated recruitment of golgins and GARP to the TGN.** This model is based on results shown in this article and previous publications cited in the text. The multispanning membrane protein SYS1 recruits ARFRP1 to the TGN, possibly by acting as a GEF that converts ARFRP1-GDP to ARFRP1-GTP. This process involves the N-terminally acetylated amphipathic α-helix of ARFRP1. ARFRP1 then promotes the recruitment and/or activation of both ARL1 and ARL5 to the TGN. ARFRP1 could do so either by acting as a GEF or by recruiting specific GEFs for ARL1 or ARL5. The resulting ARL1-GTP and ARL5-GTP associate with the TGN membrane via their N-terminally myristoylated amphipathic α-helices. ARL1 in turn recruits three golgins to the TGN (Golgin-245, Golgin-97, and GCC88), which capture retrograde transport carriers containing specific cargos. ARL5, on the other hand, recruits the GARP complex. The golgins then undergo a conformational collapse (Cheung et al., 2015) that brings the carriers close to the TGN, enabling the transfer of the carriers to GARP. Finally, GARP promotes the assembly of the trans-SNARE complex that allows fusion of the carrier and TGN membranes, resulting in delivery of the specific cargos to the TGN. SYS1 and ARFRP1 thus function upstream of two other small GTPases, enabling the recruitment of distinct types of tethering factors to the TGN. A fourth golgin, GCC185, is recruited to the TGN by RAB6. It remains to be determined if and how RAB6-GCC185 coordinates with ARFRP1-ARL5-GARP to tether transport carriers to the TGN.

